# Challenges of delivering a lifestyle intervention before surgery in multiple surgical specialties in the NHS: experience from the INSPIRE trial

**DOI:** 10.1186/s13063-026-09757-6

**Published:** 2026-05-08

**Authors:** Emma Bridgeman, Chloe Beard, Katherine Joyce, Denny Levett, Dawn Phillips, Maria Pufulete, Lucy Culliford

**Affiliations:** 1 Bristol Trials Centre, University of Bristol, Bristol, UK; 2 University Hospital Southampton NHS Foundation Trust, Southampton, UK; 3 Bristol Heart Institute, University of Bristol, Bristol, UK

**Keywords:** Randomised controlled trial, COVID-19 pandemic, Pre-operative lifestyle intervention, Inspiratory muscle training

## Abstract

This paper describes the challenges of undertaking a trial of a pre-operative lifestyle intervention in multiple surgical specialties, the INSPIRE trial, and additional challenges of doing so during the COVID-19 pandemic. The INSPIRE trial was a randomised controlled trial to test if inspiratory muscle training could reduce the incidence of postoperative pulmonary complications in patients undergoing cardiac, thoracic and abdominal elective surgery. We present the challenges of trial conduct alongside the adaptations introduced to mitigate the impact of those challenges. The INSPIRE trial was halted by the funder at the end of the internal pilot phase. We were therefore unable to assess the effect of some of the adaptations, and there were further adaptations we were unable to make. We are using our experience to make recommendations for future similar trials.

## Background

The INSPIRE trial was designed to investigate the effectiveness of a prehabilitation intervention, pre-operative inspiratory muscle training (IMT), for reducing postoperative pulmonary complications (PPC) in participants at high risk of having lung complications after major surgery (ISRCTN10644366) [1]. INSPIRE was a randomised controlled trial with three groups: high-resistance IMT (intervention: breathing exercises with a device), ‘sham’ IMT (control: low-resistance breathing exercises with a device) and usual care (control), randomised in a ratio of two:one:one. Participants were recruited and randomised whilst on the waiting list for elective surgery in a range of specialties, and the intervention and sham control breathing exercises were conducted at home for 2 to 8 weeks prior to admission for surgery. Delivering a prehabilitation intervention before surgery in hospitals came with many challenges, compounded by the COVID-19 pandemic.

The trial aimed to recruit 2500 participants but was closed by the funder as part of the NIHR RESET initiative [2] after completion of the internal pilot due to concerns that the trial would not be able to recruit the full target sample size in a timely fashion. Although participants and staff reported that the trial was acceptable.

The trial opened to recruitment in December 2019, shortly before the disruption to and subsequent reduction in elective surgery due to the COVID-19 pandemic that began in early 2020. Adaptations to the trial were made and recruitment to the internal pilot was achieved (*n* = 320 participants) although this took 29 months rather than the predicted 8 months and the trial was closed by the funder. This paper outlines some of the challenges faced by the trial, many of which are not related to the pandemic and relevant to clinical trials of lifestyle interventions or trials aiming to include multiple surgical specialties. We also present adaptations or mitigations that were put in place to overcome these challenges. A summary of the challenges, adaptations and recommendations is presented in Table 1.

**Table 1:** A summary of the challenges, how these were mitigated in INSPIRE and advice for future trials of lifestyle interventions in surgical patients

Challenge	How we mitigated challenge	Considerations for future trials
**Trial related**		
Blinding of participants and staff not involved in intervention training/delivery	Larger site teams Data collection forms split into blinded/unblinded with associated restricted database access Unblinding CTU staff to facilitate oversight	Consider resource implications Direct data entry at site (to prevent unblinding via sight of completed paper data collection forms) Use electronic PROMs or independent administrative staff to collect PROMs Modular database design and separate training session for blinded/unblinded staff
Recruiting participants from multiple specialties per site	Treat each specialty as a site each with a PI	Provide trial resources (e.g. equipment) to each specialty Recruitment projections to be conservative and not assume that all specialties will take part in all sites
NHS indemnity and ownership of the IMT devices	IMT devices were gifted to NHS Sponsor	Devices and other equipment to be purchased by NHS Sponsor or sites
Staff and research time	CTU assisted with site activities such as data entry	Plan and resource appropriately any activities that could be carried out by the CTU on behalf of the site Engage with staff not actively engaged with research early to facilitate GCP and other general research training
Data collection for outcome measures and resource use	Contracts for data sharing with private hospitals that carried out procedures on behalf of participating NHS sites	Use of routinely collected data such as Hospital Episode Statistics Consider payments for information provision form primary care providers
Collecting data on the day of surgery		Consider using a PROM that can be completed at home prior to admission (if appropriate)
**COVID-19 pandemic related**		
Face-to-face appointments	Protocol amendment to allow remote participant contact Infection control procedure developed for IMT as an aerosol generating procedure	Flexible protocol that allows a range of contact options Use devices that allow data on participant usage to be downloaded

## Challenges, impact and adaptations

### Blinding

The participants in the usual care (control) arm and those randomising and delivering training and support to participants could not be blinded due to the nature of the intervention and control procedures. However, participants randomised to IMT and allocated IMT or sham IMT were blinded to IMT group allocation. In order to blind outcome assessments, the clinical care team (i.e. surgeon, anaesthetist and post-operative care team) and all members of the research team not involved in randomising or administering the IMT interventions were blind to participants’ allocation. This meant that participating sites needed teams with blinded and unblinded personnel. This required bigger teams at sites to deliver the trial. It also meant that any staff unavailability or turnover could easily prevent tasks that were assigned only to blinded or unblinded staff from being completed.

This trial feature added complexity to site initiation training, necessitating a full training session for all site staff and separate device training sessions for the unblinded team members. These training sessions were completed face-to-face before the COVID-19 pandemic, as this was considered the best way to train staff to use the IMT devices. However, this required a large time commitment from the trainers (including the trial managers and staff from the device manufacturer) and were difficult and time consuming to arrange. These training sessions were moved online and completed via videocalls during the COVID-19 pandemic which required less time commitment from the trainers as no travel was needed, it also allowed sessions to be split to make it easier for site staff to attend. As far as possible, training over video call mimicked face-to-face training by including verbal instructions, a demonstration by the trainer who then watched the trainee’s technique and gave advice for improvement until they appeared to be using optimal technique. However, online training may still have compromised the quality of the training in the use of the IMT device.

Processes were put in place to facilitate maintaining the blind within site teams. For instance, in order to facilitate blinded data collection, the database and paper data collection forms were split into blinded and unblinded sections. This made the set-up of the database more complex with careful specification of roles and access rights needed, and extra testing required compared to a database where all site staff have the same level of access. Care was also taken to direct data queries to the correct members of the site team which required more time and resources from the central trial management team at the Clinical Trials Unit (CTU). Sites needed to be able to securely store the blinded and unblinded paper data collection forms in separate locations, which meant they had to find additional secure storage options. At the time, direct data entry was not offered; however, for future trials, this would overcome this latter problem if paper data collection forms had not been used.

The trial management team at the CTU responsible for day-to-day management of the trial also sent out the follow-up questionnaires to participants or completed them by phone with participants who had not returned the postal questionnaire, and for this reason the plan was to keep them blinded to allocation. However, it became apparent that this hampered their ability to deal with queries from sites and participants. CTU staff were also not able to have any oversight of the data entered or the queries on the unblinded database forms, which meant it was impossible to know if there were any significant issues related to the intervention. After assessing the risk to the trial from unblinding versus the risk from a lack of oversight, the decision was made to unblind the trial management (TM) team. The risk of the TM team being able to introduce any bias in the completion of questionnaires was judged to be low compared with the risk to the integrity of the trial if they were unable to maintain effective oversight and deal with any issues in intervention delivery and adherence and data collection as they arose. For future trials, a solution would be to budget for follow-up to be conducted by blinded administrative staff not involved in the day-to-day running of the trial. More resources at the CTU would also allow a separation of blinded and unblinded CTU staff for other tasks if needed. Use of electronic patient-reported outcome measures (PROMs) would also prevent any risk of bias that may be introduced by unblinded staff completing questionnaires by phone.

#### Recruiting participants from multiple specialties per site

The intervention tested in INSPIRE is applicable to multiple surgical specialties; therefore, to ensure the generalisability of trial results, the trial was designed to recruit participants from a range of surgical specialties (cardiac, thoracic and abdominal). We followed the usual practice of assigning one Principal Investigator (PI) per site, and this PI was responsible for all research activity at the site and across all specialties. Some PI responsibilities, such as signing off entries on a site delegation log or assessing safety events needed to be delegated to a suitably qualified person within the specialties who knew the staff members and had the relevant clinical expertise. However, as these ‘sub-PIs’ could not be officially named as site PI, they appeared to be less invested in the trial and this is likely to have led to reduced recruitment in their specialty. Engagement activities such as investigator meetings and newsletters with recruitment league tables did not help. A further solution to this, which we started to implement shortly prior to the closure of the trial, was to treat each specialty as a site, so that each specialty had a site PI who was fully invested in the trial. We were not able to assess if this change had any impact, but are planning to implement this in other trials with similar cross-specialty research activities. The Associate PI scheme was not available during this trial; however, given the success of this scheme since its inception, it is reasonable to assume that it would have been helpful. We have had success in using the scheme for more recent trials that involve more than one clinical specialty and would recommend that others do so.

Some members of the site research teams, for example physiotherapists, were not specific to a surgical specialty and covered multiple specialties in different departments, usually located in separate areas of the hospital, different buildings or even different hospitals for large National Health Service (NHS) Trusts. This made the logistics of running the trial harder. The research team needed to be in the right place at the right time which may have been different for each specialty/participant, and they needed access to the study laptop and training device in order to deliver training in the intervention and conduct assessments. This could be addressed by procuring and providing additional equipment, although this has a cost implication, or increasing the number of such staff at each site. However, this relies on enough research capacity within these staff groups which is outside of the control of the Chief Investigator and CTU. Research capacity for non-COVID-19 research was severely impacted during the pandemic and had not recovered by the time the funding was halted for this study.

Lastly, when designing this trial and calculating the number of sites needed and the time needed to recruit the target sample size, it was assumed all sites would recruit to all three specialties. However, some sites only opened to recruitment in one or two specialties and this meant more sites were needed than originally planned and recruitment was slower than predicted. Each site required at least one trial-provided laptop to link with the IMT devices to obtain data from them, and the budget included per-site payments for site set-up and close-down, in addition to per-participant payments. If the trial had proceeded beyond the internal pilot, additional funds would have been needed to overcome this. If a trial recruiting from multiple specialties is planned, it would be prudent to assume that sites only recruit in one or two specialties to make sure that funds and resources are available for enough sites.

#### NHS indemnity and ownership of the IMT devices

Participants who were randomised to IMT were given an IMT device to take home by their recruiting site, and each site also had a training device and a laptop. For ease, the University that hosts the CTU purchased the devices so that the central TM team could provide devices to sites on site initiation. However, any equipment used in NHS hospitals requires oversight by the Medical Equipment Management Organisation team. They were unable to accept University-owned devices onto their inventory, and NHS indemnity could not be applied to them. Lengthy contract negotiations took place between the University who owned the devices and the NHS Trust sponsoring the research, which led to at least a 6-month delay in set-up of the trial. The solution was for the devices to be gifted to the Sponsor. To avoid similar issues, it is advised that any devices or equipment intended for use at NHS sites be purchased via an NHS organisation.

#### Staff and research time

Sites that used physiotherapists or exercise physiologists to deliver training for the IMT to participants had difficulties managing research activities alongside routine clinical workloads. This was because the physiotherapists or exercise physiologists did not have dedicated research time allocated to them. As a result, they were often unable to complete all data collection tasks such as entering data into the database from completed paper data collection forms. These tasks were completed on their behalf by the central TM team at the CTU, creating unplanned work for the CTU. This also increased the time it took for any resultant data queries to be resolved as the central TM team could not address the queries as they arose and had to raise the queries with the local site team for resolution.

Some physiotherapists had not completed NIHR Good Clinical Practice (GCP) training prior to participating in this trial as they had not previously undertaken research activities. This required extra time for training in research skills and GCP, and although this provided a good opportunity to link them up with their local allied health professional champions, it was again not a task the CTU expected to deliver. The physiotherapists or exercise physiologists were often not based in the same department as the other members of the research team. Together with their limited capacity to undertake research, this limited sites’ ability to recruit participants.

Increasing the involvement of these allied health care professionals more widely in research, providing dedicated research time and facilitating GCP and generic research training would help overcome these barriers.

#### Face-to-face appointments

IMT training is routinely delivered in person, and the trial design investigated whether a face-to-face follow-up visit was needed to support participants in using the IMT device correctly. During the COVID-19 pandemic, national advice was to avoid face-to-face visits that were solely for research purposes, and the use of online follow-up appointments was introduced to the trial design. This change led to a redesign of some of the INSPIRE trial conduct to allow remote contact with participants, requiring a protocol amendment. A consideration for future studies is to make sure that, where appropriate, the protocol is sufficiently flexible to allow a range of options for participant contact.

IMT was also classed as an ‘aerosol generating procedure’ and a bespoke risk assessment was conducted to reduce the risk of COVID-19 infections when face-to-face visits resumed. The infection control team at the lead site developed a process for face-to-face visits; this required the room in which IMT was conducted to be ventilated for 45 min before another participant could be seen in the same room. This process had to be adopted by each site’s infection control team which caused further delays in restarting recruitment following a temporary halt to recruitment during the peak of the pandemic. Implementation of the process also had resource implications for sites and reduced the rate of recruitment they could achieve as it reduced the number of patients that could be seen in a day.

Review by site teams of the participants’ adherence to the intervention, assessment of participant ‘progress’ with the breathing exercises (increasing the load in the IMT device) and collection of data stored in the IMT devices were also hampered by remote follow-up. In order to assess adherence to the breathing exercise protocol and appropriate increase of the training load, participants used paper diaries to record their sessions and specific data relating to the intensity of the training from the device. Although the diaries could be returned to site staff after the intervention was completed, any real-time assessment of compliance required the participant to read out their diary entries over the phone to the staff member. This was time-consuming and often not feasible as it included many data items collected over a 2-week period. Using devices where data could be downloaded remotely would have helped mitigate against this issue; however, this functionality was not available.

#### Data collection for outcome measures and resource use

Data collection included admissions to hospital after discharge following the index operation to provide data for the primary outcome (post-operative pulmonary complications) and resource use data for the health economic evaluation. Participants who experienced re-admissions were often not admitted to the hospital where they were recruited from, and consent covered data access to their medical records. This is because cardiac, thoracic and abdominal surgery is undertaken at tertiary centres and participants attend for surgery from a very large geographical area and are likely to attend a different hospital near to where they live for any subsequent admissions. In these cases, staff at trial sites found it very difficult and time consuming to obtain information about the admission from the admitting hospital, usually by telephone. Furthermore, if a participant did not return their follow-up questionnaire (detailing if they had been admitted to hospital), the trial site would not know if an admission had taken place. Occasionally primary care staff could provide the necessary information but often did not have capacity to deal with these requests. A potential solution would be to use routine data, currently provided by NHS England. However, Hospital Episode Statistics (HES) data provided by NHS England were unlikely to provide the detail needed to confirm if a primary outcome event had occurred. A single NHS electronic health record would also make follow-up across tertiary and secondary care settings more efficient.

Some participants had their operations at private hospitals either by choice or through NHS waiting-list initiatives. To maximise data completeness, contracts were arranged by and put in place between the trial (NHS) sites and the private hospitals to allow access to records to collect trial data about the operation and post-operative recovery prior to discharge, which could include primary outcome events.

#### Collecting data on the day of surgery

The rationale for the trial was that IMT would strengthen the inspiratory muscles in the chest prior to surgery to improve post-operative pulmonary outcomes. The strength of the inspiratory muscles is measured by the Maximal Inspiratory Pressure (MIP). Measurements of MIP were scheduled for baseline and on the day of surgery to measure how effective IMT had been in the pre-operative period. However, often participants were unable to be seen on the day of their operation, meaning many day of surgery measurements could not be taken. This was due to a variety of reasons such as staff shortages, shift start and finish times, surgery being moved at late notice, and day of surgery admissions where participants were admitted early in the morning (typically 6–7am) for an 8am theatre slot. This issue is a common challenge with pragmatic trials in hospital-based settings, and contact with participants and completing data collection on day of surgery are always difficult. A solution would be to ask participants to attend the day before their operation for measurements (sometimes facilitated by transfer/admission the afternoon/evening prior to their operation), but if this is not part of their usual care pathway, this is resource intensive and inconvenient for the participant.

Another major challenge when delivering a prehabilitation intervention before surgery is managing the unpredictable elective surgery lists, which impacts not only recruitment but also participant retention and adherence with the intervention. The trial was designed for the intervention to be delivered for a maximum time of 8 weeks, but waiting times increased during the COVID-19 pandemic and many participants waited much longer for their operation. The challenge for site staff was encouraging participants to continue their assigned breathing exercises and also knowing when to instruct participants to begin recording their IMT exercises pre-surgery into their diaries. A minimum of 2 weeks of data into the diaries was requested from each participant, and ideally this would be the last two weeks of their training period. If operations were delayed, we wanted to avoid asking participants to complete diaries for an extended period, which could lead to less motivation to do so and risk data integrity. However, operations were often postponed just days before the planned operation date, so it was difficult for the trial team to optimise when to instruct dairy completion. Some participants also never underwent surgery before the trial ended and no surgery data was collected. In an attempt to collect some data on these randomised participants they were then given the opportunity to be followed up despite not undergoing surgery. However, some of these participants could not be contacted despite multiple attempts, possibly due to dissatisfaction that their operation had not taken place.

## Conclusion

Despite being closed to recruitment at the end of the internal pilot, the INSPIRE trial offers many insights into the challenges of conducting research during a pandemic, and also in research investigating a pre-operative lifestyle intervention in multiple surgical specialties. Some mitigations and adaptations were put in place, and trials with similar designs or settings should consider adopting these into their design and conduct, and in some cases these adaptations have become common design features in trials designed since the COVID-19 pandemic. Other challenges that could not be addressed by the INSPIRE team provide pointers for those designing future research to consider how they might mitigate against similar issues.

## Data Availability

Not applicable, trial data was not used for this commentary piece.

## Abbreviations

CTU: Clinical Trials Unit
GCP: Good Clinical Practice
IMT: Inspiratory muscle training
MIP: Maximal inspiratory pressure
NHS: National Health Service
PPC: Postoperative pulmonary complications
PROMs: Patient-reported outcome measures
TM: Trial management
